# Vitamin D levels correlate with lymphocyte subsets in elderly patients with age-related diseases

**DOI:** 10.1038/s41598-018-26064-6

**Published:** 2018-05-16

**Authors:** Xudong Mao, Bin Hu, Zhiwen Zhou, Xubin Xing, Yan Wu, Jing Gao, Yue He, Ying Hu, Qihong Cheng, Qing Gong

**Affiliations:** 1 Department of Geriatrics, Zhongshan-Xuhui Hospital, Shanghai Clinical Center, Chinese Academy of Sciences, Shanghai, China; 2 Department of Respiratory, Zhongshan-Xuhui Hospital, Shanghai Clinical Center, Chinese Academy of Sciences, Shanghai, China; 3 Department of Cardiology, Zhongshan-Xuhui Hospital, Shanghai Clinical Center, Chinese Academy of Sciences, Shanghai, China; 4 Department of Cardiology, Ruijin Hospital Luwan Branch, Shanghai Jiaotong University School of Medicine, Shanghai, China; 5 Department of Psychiatry, Shanghai Mental Health Center, Shanghai, China; 6 Department of Information, Zhongshan-Xuhui Hospital, Shanghai Clinical Center, Chinese Academy of Sciences, Shanghai, China

## Abstract

Hypovitaminosis D is associated with age-related illnesses, including hypertension, cardiovascular disease (CRVD), cerebrovascular disease (CAD) and type 2 diabetes mellitus (T2DM). In our retrospective observational study, blood samples of elderly healthy controls (n = 461) and patients with age-related diseases (n = 8,621) were subjected to flow-cytometry in order to determine correlations between age-related diseases and cluster of differentiation 4 (CD4), CD8, CD3, and CD19 lymphocyte markers, as well as serum levels of 25-hydroxyvitamin D_2_ (25(OH)D_2_) and 25-hydroxyvitamin D_3_ (25(OH)D_3_). More than 70% of the patients in each disease group had total vitamin D < 20 ng/mL (*P* < 0.001). In CRVD patients, CD3 and CD19 correlated (*P* < 0.05) with 25(OH)D_3_. In CAD patients, CD8, CD4, CD19 and CD4/CD8 correlated (*P* < 0.05) with 25(OH)D_2_, and CD8 correlated (*P* < 0.05) with 25(OH)D_3_. In T2DM and hypertension patients, CD8, CD3, CD19 and CD4/CD8 correlated with 25(OH)D_3_. Progressive trends (*P* < 0.05) towards increased CD8 and CD4/CD8 were observed in vitamin-D-deficient T2DM and hypertension patients. Significant differences (*P* < 0.05) in CD8 were observed in vitamin-D-deficient CAD patients, whereas significant differences (*P* < 0.05) in CD8 and CD19 were observed in CRVD patients. Higher CD8 and CD4/CD8 in 25(OH)D-deficient T2DM and hypertension patients suggested a Th1 lymphocyte profile induction. Increases in CD8-positive lymphocytes suggested a similar, less pronounced effect in vitamin-D-deficient CRVD and CAD patients.

## Introduction

Vitamin D refers to a group of lipid-soluble compounds that act as precursors for the production of the biologically active secosteroid, 1,25-dihydroxyvitamin D, which is primarily produced in the kidney^1^. Vitamin D is known to function in a wide range of homeostatic processes including calcium absorption and bone remodeling^2^. The major vitamin D metabolites in serum are 25-hydroxyvitamin D_2_ (25(OH)D_2_) and 25-hydroxyvitamin D_3_ (25(OH)D_3_), with the serum level of 25(OH)D_3_ most often much higher than the level of 25(OH)D_2_^3,4^. The dermal 25(OH)D_3_ conversion after exposure to solar radiation serves as the major source of vitamin D^5^, whereas 25(OH)D_2_ is present in relatively few foods including egg yolk, fish and mushrooms and is used to fortify mainly milk, orange juice and cereals. Recent research has revealed alternative vitamin 3 pathways leading to various (OH)D_3_ products other than 25(OH)D_3_ in skin, placenta and porcine adrenal gland^6–8^.

Vitamin D deficiency is a global pandemic^3,9^ and observational studies based on 25(OH)D concentrations revealed beneficial effects for patients with a variety of illnesses^10,11^ including metabolic syndrome (MetS)^12,13^, obesity^9,14^, certain cancers^15,16^, a number of autoimmune disorders^15,17^, hypertension^18,19^, cardiovascular disease (CVD)^20^, and T2DM^21,22^. However, whether vitamin D deficiency contributes to the pathology of these diseases remains largely unclear.

Investigations worldwide have shown that vitamin D levels wane in the elderly^23,24^, with women often exhibiting vitamin D deficiency at younger ages than men^25^. Serum markers of both innate and adaptive immunity are associated with the vitamin D status^26,27^. It has been demonstrated that 1,25(OH)2D_3_, the most biological active metabolite of vitamin D, acts on T and B lymphocytes to modulate both the cytotoxic and antibody-producing functions of lymphocytes^28,29^. Previous studies have shown that 1,25(OH)2D_3_ synthesis could activate innate immune responses, while suppressing adaptive immune responses^30,31^. A decreased CD4+/CD8+ ratio, resulting from reduced CD4+, was reported in patients with acquired immune deficiency syndrome (AIDS), suggesting CD4+/CD8+ reversion was an important indicator of immunodeficiency diseases and viral infection^32^. Similarly, T cell variations have been correlated with cancer outcomes^33,34^ and T2DM^35^.

Vitamin D deficient elderly persons frequently exhibit a progressive increase in serum levels of acute-phase reaction proteins and proinflammatory cytokines^36–39^, a set of conditions associated with chronic diseases that are highly prevalent in the elderly, including CVD, neurodegenerative diseases and glucose metabolism disorders^40,41^. Animal model and cell culture studies have demonstrated beneficial immunomodulatory effects of vitamin D, which is thought to induce an immunological transition from a Th1/Th17 response to a Th2/T_reg_ response based on the cytokine profiles of immune cell types measured following vitamin D treatment^36,42^.

Observational studies of elderly people in Italy and Ireland found that the serum level of IL-6 was inversely associated with the vitamin D status^36,37^. Blockade of the IL-6 receptor with tocilizumab has been shown to suppress Th1 and Th17 signaling and stimulate beneficial Th2 responses in patients with early-stage rheumatoid arthritis^43^, and suppression of Th17 signaling with the vitamin D receptor agonist, elocalcitol, was shown to reduce autoimmunity in non-obese diabetic mice^44^. Studies of immune cells have also shown that treatment with 1,25(OH)_2_D_3_ reduced the expression of IL-6 and various other proinflammatory factors^45^. Given the high prevalence of elevated serum IL-6 in vitamin D deficient elderly persons, we retrospectively analyzed the records of a large population of elderly Chinese people to investigate whether the immune marker profiles of lymphocyte subsets correlated with vitamin D levels in patients with illnesses more common diagnosed in the elderly such as T2DM, cerebrovascular disease (CRVD), coronary artery disease (CAD), hypertension and cancer^46^.

## Subjects and Methods

### Patients

We analyzed the records of patients’ ≥40 years of age who were treated for various conditions at the Hospital of Shanghai Zhongshan-Xuhui between May 2012 and August 2016. Patients with hepatic failure, serum creatinine >120 µmol/L, hyperthyroidism, hypothyroidism, immune disorders or infection were excluded from our study. Patients receiving hormone medication were also excluded from our analysis. A total of 461 healthy volunteers (control subjects) were also enrolled in our study. Informed written consent was obtained from all of the patients and control subjects prior to their participation in our study. The study was approved by the Institutional Review Board of ZhongShan-Xuhui Hospital, and was carried out in accordance with the principles of the Declaration of Helsinki for ethical research involving human subjects.

### Measurement of vitamin D metabolites

Serum samples collected from patients and healthy volunteers were subjected to liquid chromatography-tandem mass spectrometry (LC-MS/MS) using deuterated 25(OH)D_2_ (6,19,19-d_3_) and deuterated 25(OH)D_3_ (26,26,26,27,27,27-d_6_) (Sigma-Aldrich, St. Louis, MO, USA) as internal standards for 25(OH)D_2_ and 25(OH)D_3_, respectively. The analysis was performed using an API 4000 LC-MS/MS system (AB Sciex Pte, Framingham, MA, USA) equipped with a Shimadzu liquid chromatograph (Kyoto, Japan). The accuracy of low, medium, and high concentration controls was 85–115%, and the precision (CV) was <15%. The inter- and intra-assay coefficients of variation were <10%. Analyst 1.5 software (Applied Biosystems, Foster City, CA, USA) was used for data collection and quantitative analysis.

### Measurement of immunity-related indexes

CD3 represents the total T cell number (thymus dependent lymphocyte) and CD19 is a cell surface receptor complex component that regulates B lymphocyte responses^47^. Cells from whole blood samples were subjected to flow cytometry analysis to determine the proportions of lymphocyte subsets in peripheral venous blood within 2 h of collection, based on binding of the following monoclonal antibodies (mAbs): Anti-CD4, anti-CD8, anti-CD3, anti-CD56 and anti-CD19 (BD Biosciences, San Jose, CA, USA), The stained cells were sorted using a FACS Aria flow cytometer (BD Biosciences) using appropriate isotype controls for gating, and the data were analyzed using FlowJo software (Tree Star, Ashland, OR, USA).

### Statistical analysis

All statistical analyses were performed using SPSS, version 18.0 (IBM, Armonk, NY, USA). Continuous variables are presented as the median ± interquartile range (IQR). Differences between patients with different diseases were compared using variance analysis. Categorical variables were summarized as a percentage of the whole and a chi-squared test was used to detect differences. Pearson correlation analysis was used to evaluate the correlations between indicators in two different groups, with *P* < 0.05 indicating a statistically significant difference between the two groups.

## Results

### Patient characteristics

Demographic and clinical data for the patients and control subjects are presented in Table 1. The healthy control subjects included 256 men and 205 women with a mean age of 76 ± 15 years. A total of 8,621 patients (4,215 men and 4,406 women) with a mean age of 80 ± 13 years (range: 40–106 years) were included in our analysis. The most common diagnoses for these patients included the following categories in descending order of prevalence: CRVD (32.2%, n = 2,775); CAD (25.2%, n = 2,174); T2DM (17.5%, n = 1,512); and hypertension (15.0%, n = 1,292). Other diagnoses accounted for 10.1% of the study population, and included digestive disease (n = 143), kidney disease (n = 141), malignancy (n = 111), fracture (n = 87), osteoporosis (n = 40), MetS (n = 36), liver disease (n = 23), gout (n = 12), anemia (n = 7), and other diseases (n = 268).

**Table 1:** Comparison of Vitamin D and immunity-related indexes between healthy volunteers (N = 461) and patients with various diseases (N = 8,621).

Patient group	Men (n[%])	Women (n[%])	Mean age ± SD (y)	Total 25(OH)D (ng/mL)	25(OH)D_3_ (ng/mL)	25(OH)D_2_ (ng/mL)	CD4 (%)	CD8 (%)	CD4/ CD8	CD3 (%)	CD19 (%)	CD56 (%)
Healthy Control	256 (55.5)	205 (44.5)	76 ± 15	18 ± 9	18 ± 9	1.5 ± 2.5	45 ± 5.0	27 ± 6.0	2.0 ± 0.40	69 ± 8.0	13 ± 5.0	17 ± 6.0
CRVD (N = 2,775)	1,469 (52.9)	1,306 (47.1)	75 ± 20	15 ± 9	13 ± 8	0.6 ± 1.6	45 ± 12	23 ± 11	2.0 ± 1.3	71 ± 12	10 ± 7.0	16 ± 11
CAD (N = 2,174)	1,109 (51.0)	1,065 (49.0)	87 ± 8	16 ± 10	13 ± 9	1.0 ± 2.3	43 ± 15	24 ± 13	1.8 ± 1.4	71 ± 14	9.0 ± 8.0	17 ± 13
T2DM (N = 1,512)	709 (46.9)	803 (53.1)	74 ± 18	16 ± 11	15 ± 10	0.5 ± 1.4	45 ± 13	22 ± 11	2.0 ± 1.4	71 ± 12	11 ± 6.5	16 ± 11
Hypertension (N = 1,292)	542 (42.0)	750 (58.0)	81 ± 18	17 ± 10	15 ± 10	0.7 ± 1.7	45 ± 13	22 ± 11	2.0 ± 1.5	70 ± 12	11 ± 8.0	16 ± 12
Digestive disease (N = 143)	66 (46.2)	77 (53.8)	73 ± 24	16 ± 12	14 ± 11	1.0 ± 1.6	45 ± 15	22 ± 12	1.9 ± 1.5	71 ± 14	12 ± 7.0	16 ± 16
Kidney disease (N = 141)	77 (54.6)	64 (45.4)	77 ± 16	12 ± 9	9.2 ± 7	1.3 ± 2.4	42 ± 17	24 ± 13	2.0 ± 1.4	71 ± 15	7.0 ± 7.0	18 ± 13
Malignant tumor (N = 111)	67 (60.4)	44 (39.6)	81 ± 21	12 ± 11	11 ± 10	0.8 ± 2.4	38 ± 16	27 ± 17	1.6 ± 1.3	71 ± 18	7.0 ± 9.0	17 ± 17
Fracture (N = 87)	32 (36.8)	55 (63.2)	65 ± 21	18 ± 12	16 ± 10	0.6 ± 1.7	45 ± 12	24 ± 12	1.8 ± 1.1	72 ± 13	12 ± 6.0	12 ± 13
Osteoporosis (N = 40)	5 (12.5)	35 (87.5)	72 ± 21	20 ± 12	20 ± 14	1.2 ± 2.1	46 ± 11	23 ± 13	2.1 ± 1.8	68 ± 10	10 ± 7.5	15 ± 15
MetS (N = 36)	15 (41.7)	21 (58.3)	68 ± 15	24 ± 10	23 ± 9	0.04 ± 1.5	45 ± 8.0	22 ± 8.5	2.1 ± 1.1	70 ± 9.5	13 ± 6.0	15 ± 10
Liver disease (N = 23)	18 (78.3)	5 (21.7)	82 ± 33	11 ± 7	11 ± 7	0.03 ± 1.1	36 ± 6.0	20 ± 19	1.8 ± 1.3	61 ± 24	11 ± 16	22 ± 16
Other disease (N = 268)	94 (35.1)	174 (64.9)	66 ± 23	16 ± 10	15 ± 9	0.6 ± 1.8	44 ± 13	23 ± 10	1.8 ± 1.4	72 ± 12	12 ± 8.0	14 ± 9.5
Gout (N = 12)	9 (75.0)	3 (25.0)	84 ± 13	14 ± 9	11 ± 8	1.2 ± 1.6	39 ± 13	27 ± 15	1.4 ± 1.2	67 ± 6.0	9.0 ± 9.0	20 ± 14
Anemia (N = 7)	3 (42.9)	4 (57.1)	83 ± 15	12 ± 8	12 ± 9	0.5 ± 1.6	45 ± 15	16 ± 11	2.5 ± 2.9	70 ± 19	15 ± 14	16 ± 10

Data are presented as the number of patients and percentage of the patient group [n(%)] or median ± inter quartile range.

*Sum of 25(OH)D_3_ and 25(OH)D_2_ levels.

SD, standard deviation; CRVD, cerebrovascular disease; CAD, coronary artery disease; T2DM, type-2 diabetes; MetS, metabolic syndrome; 25(OH)D_2_, 25-hydroxy vitamin D_2_; 25(OH)D_3_, 25-hydroxy vitamin D_3_.

Only the MetS and osteoporosis groups had a total serum level of vitamin D within the reference range (20–30 ng/mL), the latter of which was likely due to the regular use of vitamin D dietary supplements. Total serum vitamin D in the healthy control group was slightly below the reference range lower limit^48^, which is consistent with the findings of previous studies of the prevalence of vitamin D deficiency in the elderly^49,50^. The 25(OH)D_3_ and 25(OH)D_2_ serum concentrations of healthy control subjects differed significantly compared to some patients (Table 1).

### Correlations between vitamin D levels and lymphocyte subsets

We investigated the relationships between lymphocyte immune markers and the serum levels of 25(OH)D_2_ and 25(OH)D_3_ in the various disease groups using correlation analysis, and the results of the analysis are presented in Table 2. In CRVD patients, we observed a negative correlation between CD3 and 25(OH)D_3_ and positive correlations between CD19 and 25(OH)D_3_ and CD56 and 25(OH)D_2_. Given that 25(OH)D_3_ is the greater contributor to vitamin D status, these results suggest that vitamin D deficiency might correlate with a shift from humoral immunity to cell mediated immunity in CRVD patients. However, the lack of significant correlations for CD4, CD8, and CD4/CD8 in CRVD patients as well as the missing significance of total 25(OH)D clearly limit such an interpretation.

**Table 2:** Analysis of correlations between 25(OH)D_2_ and 25(OH)D_3_ levels and lymphocyte subsets in elderly patients with age-related illness.

Patient group	Immune marker	25(OH)D_3_		25(OH)D_2_		Total 25(OH)D	
		*r*	*P*	*r*	*P*	*r*	*P*
CRVD (N = 2,775)	CD8	−0.04	0.03	−0.00	0.98	−0.04	0.04
	CD19	0.04	0.04	−0.04	0.05	0.02	0.25
CAD (N = 2,174)	CD8	−0.05	0.03	0.13	<0.001	0.03	0.14
	CD4	0.02	0.25	−0.07	0.001	−0.02	0.42
	CD19	0.00	0.97	−0.09	<0.001	−0.05	0.03
	CD4/CD8	0.03	0.16	−0.04	0.045	0.003	0.87
T2DM (N = 1,512)	CD8	−0.13	<0.001	−0.02	0.52	−0.13	<0.001
	CD3	−0.09	0.001	−0.002	0.95	−0.09	0.001
	CD19	0.08	0.002	−0.01	0.75	0.08	0.003
	CD4/CD8	0.06	0.01	0.01	0.66	0.07	0.01
Hypertension (N = 1,292)	CD8	−0.07	0.01	0.02	0.47	−0.06	0.03
	CD3	−0.08	0.01	0.01	0.61	−0.07	0.01
	CD19	0.12	<0.001	−0.04	0.17	0.11	0.001
	CD4/CD8	0.06	0.02	−0.00	0.95	0.06	0.03
Kidney disease (N = 141)	CD3	—	—	0.17	0.049	0.12	0.20
	CD4/CD8	0.18	0.03	—	—	0.15	0.08
Digestive disease (N = 143)	CD4	—	—	−0.29	0.001	−0.13	0.11
	CD8	—	—	0.27	0.001	0.16	0.06
Osteoporosis/fracture (N = 127)	CD8	−0.19	0.03	—	—	−0.18	0.043

Note: CD 56 was not included in the table due to missing significant correlations with 25(OH)D_2_ or 25(OH)D_3_ or total 25 (OH)D; CAD, coronary disease; CRVD, cerebrovascular disease; T2DM, type 2 diabetes mellitus; 25(OH)D_2_, 25-hydroxyvitamin D_2_; 25(OH)D_3_, 25-hydroxyvitamin.

In patients with CAD, we observed a significant positive correlation between CD8 and 25(OH)D_2_ and significant negative correlations between CD8 and 25(OH)D_3_, CD4 and 25(OH)D_2_, CD19 and 25(OH)D_2_ and CD4/CD8 and 25(OH)D_2_. However, reduced 25(OH)D_2_ significantly correlating with increased CD4, CD19, and CD4/CD8 as well as missing significance of total 25(OH)D, limits the interpretation of these results in CRVD patients.

In T2DM patients, the level of 25(OH)D_3_ negatively correlated with CD8 and CD3, and positively correlated with CD19 and CD4/CD8, which also was visible for total 25(OH)D_._ These results suggest that vitamin D deficiency correlates with the induction of Th1 immunity in T2DM patients, and that patients with T2DM might benefit from 25(OH)D_3_ dietary supplements.

In patients with hypertension, 25(OH)D_3_ and total 25(OH)D negatively correlated with CD8 and CD3 and positively correlated with CD19 and CD4/CD8, a finding which suggests that patients with hypertension might also benefit from 25(OH)D_3_ dietary supplementation.

Though there were some correlations between 25(OH)D_2_ and 25(OH)D_3_ with lymphocyte subsets, the results for kidney disease, digestive disease and fracture/osteoporosis groups were insufficient to draw any reliable inference of the effects of serum levels of vitamin D metabolites on these diseases.

### Stratified analysis of vitamin D levels in elderly patients with age-related illnesses

Based on our findings regarding correlations between the levels of vitamin D metabolites and lymphocyte subsets, we performed a stratified analysis of the relationship between immune cell markers and vitamin D in patients with the most prevalent age-related diseases represented in our study. To this end, we first performed a stratified analysis of 25(OH)D_2_ and 25(OH)D_3_ levels in the CRVD, CAD, T2DM and hypertension groups, the results of which are presented in Table 3. Patients were divided into 4 categories based on their serum 25(OH)D_3_ levels (<10, 10–19, 20–30 and >30 ng/mL), and into 3 categories based on the serum level of 25(OH)D_2_ (<1, 1–2, and >2 ng/mL). Significant differences were observed in the number of patients in the various 25(OH)D_3_ and 25(OH)D_2_ categories for all of the disease groups (Table 3). More than 70% of the patients in each disease group were vitamin D deficient (total vitamin D < 20 ng/mL), with most of the patients (>50%) in the 10–20 ng/mL 25(OH)D_3_ and <1 ng/mL 25(OH)D_2_ categories for all of the CRVD, CAD, T2DM and hypertension groups. Overall, these results are consistent with the findings of previous studies regarding the high prevalence of vitamin D deficiency in elderly persons^49,50^. These results suggest that either vitamin D deficiency contributes to the pathology of CRVD, CAD, T2DM and hypertension or alternatively that these diseases modify the maintenance of serum vitamin D levels in a similar manner.

**Table 3:** Stratified comparison of 25(OH) D_2_ and 25(OH) D_3_ in elderly patients with age-related illnesses.

Patients	25(OH)D_3_ (ng/mL)				P*	25(OH)D_2_ (ng/mL)			P*
	<10	10–19	20–30	>30		<1	1–2	>2	
	Patients [n(%)]					Patients [n(%)]			
CRVD (N = 2,775)	795 (28.6)^a^	1,500 (54.0)^b^	402 (14.5)^c^	78 (2.8)^d^	<0.001	1,601 (57.7)^a^	603 (21.7)^b^	571 (20.6)^b^	<0.001
CAD (N = 2,174)	617 (28.4)^a^	1,112 (51.2)^b^	364 (16.7)^c^	81 (3.7)^d^	<0.001	1,601 (57.7)^a^	603 (21.7)^b^	571 (20.6)^b^	<0.001
T2DM (N = 1,512)	365 (24.1)^a^	760 (50.3)^b^	307 (20.3)^a^	80 (5.3)^c^	<0.001	918 (60.7)^a^	326 (21.6)^b^	268 (17.7)^c^	<0.001
Hypertension (N = 1,292)	229 (17.7)^a^	700 (54.2)^b^	281 (21.7)^c^	82 (6.4)^d^	<0.001	714 (55.3)^a^	303 (23.4)^b^	275 (21.3)^b^	<0.001

^*^P < 0.0001 for overall comparison of vitamin D categories within the CRVD, CAD, T2DM, and hypertension groups.

a, b, c, d: Use of the same superscripted letter within a disease group indicates no significant difference (*P* ≥ 0.05) between the number of patients in those vitamin D categories, whereas different letters indicate a significant difference (*P* < 0.05) between vitamin D categories.

CRVD, cerebrovascular disease; CAD, coronary artery disease; T2DM, type 2 diabetes mellitus.

### Stratified analysis of correlations between vitamin D levels and lymphocyte subsets

To complete our study, we performed an analysis of immune cell markers and serum vitamin D levels in CRVD, CAD, T2DM and hypertension patients, that were stratified according to the categories of 25(OH)D_2_ and 25(OH)D_3_ concentrations established in the previous section.

As shown in Table 4, the distributions of the percentage of CD8-positive cells, CD19-positive cells, and CD56-positive cells in CRVD patients were significantly different across the various categories of serum 25(OH)D_3_ concentrations, whereas only the distribution of CD4-positive cells was significantly different across the various categories of serum 25(OH)D_2_ levels. The higher proportion of CD8-positive cells in 25(OH)D-deficient CRVD patients and the higher proportion of CD19-positive cells in CRVD patients with 25(OH)D_3_ levels >20 ng/mL suggests that low serum vitamin D promotes a Th1 immune marker profile in CRVD patients.

**Table 4:** Stratified analysis of correlations between 25(OH)D_2_ and 25(OH)D_3_ levels and lymphocyte subsets in elderly patients with age-related illness

Patient group	25(OH)D_3_ (ng/mL)				*p*	25(OH)D_2_ (ng/mL)			*P*	
	<10	10–19	20–30	>30		<1	1–2	>2		
**Cerebrovascular disease (N** **=** **2,775)**										
CD4 (%)	44 ± 10	45 ± 10	44 ± 10	46 ± 8	0.44	45 ± 9	46 ± 9	44 ± 10	0.04	
CD8 (%)	24 ± 9	24 ± 9	23 ± 9	21 ± 6	0.01	24 ± 9	24 ± 9	24 ± 9	0.67	
CD4/CD8	2 ± 1	2 ± 1	2 ± 1	2 ± 1	0.43	2 ± 1	2 ± 1	2 ± 1	0.93	
CD3 (%)	70 ± 10	71 ± 10	69 ± 10	69 ± 8	0.06	70 ± 9	71 ± 10	70 ± 10	0.19	
CD19 (%)	11 ± 6	11 ± 6	12 ± 8	11 ± 6	0.01	11 ± 6	11 ± 6	11 ± 6	0.82	
**Coronary artery disease (N** **=** **2,174)**										
CD4 (%)	42 ± 10	43 ± 18	43 ± 9	43 ± 9	0.36	44 ± 18	43 ± 9	43 ± 11	0.70	
CD8 (%)	26 ± 11	25 ± 11	25 ± 10	26 ± 9	0.04	25 ± 10	25 ± 10	26 ± 12	0.85	
CD4/CD8	2 ± 1	2 ± 1	2 ± 1	2 ± 1	0.13	2 ± 1	2 ± 1	2 ± 1	0.10	
CD3 (%)	70 ± 11	70 ± 11	70 ± 10	71 ± 11	0.67	70 ± 11	70 ± 10	70 ± 11	0.90	
CD19 (%)	9 ± 7	10 ± 7	10 ± 6	9 ± 7	0.75	10 ± 7	10 ± 6	9 ± 7	0.046	
**Type 2 diabetes (N** **=** **1,512)**										
CD4 (%)	44 ± 10	45 ± 10	46 ± 25	45 ± 10	0.69	45 ± 17	44 ± 9	45 ± 10	0.79	
CD8 (%)	25 ± 9	24 ± 9	22 ± 8	20 ± 8	<0.001	24 ± 9	24 ± 8	23 ± 8	0.21	
CD4/CD8	2 ± 1	2 ± 2	2 ± 1	3 ± 2	0.02	2 ± 1	2 ± 2	2 ± 2	0.36	
CD3 (%)	70 ± 10	70 ± 10	68 ± 9	68 ± 10	0.01	70 ± 10	70 ± 9	69 ± 9	0.82	
CD19 (%)	11 ± 6	12 ± 6	12 ± 6	12 ± 6	0.16	11 ± 6	12 ± 6	11 ± 6	0.78	
**Hypertension (N** **=** **1,292)**										
CD4 (%)	44 ± 9	45 ± 9	45 ± 9	47 ± 11	0.001	44 ± 9	45 ± 9	44 ± 9	0.35	
CD8 (%)	24 ± 9	23 ± 8	22 ± 9	22 ± 8	0.02	23 ± 8	24 ± 9	23 ± 9	0.17	
CD4/CD8	2 ± 1	2 ± 1	2 ± 1	2 ± 1	0.03	2 ± 1	2 ± 1	2 ± 1	0.72	
CD3 (%)	70 ± 10	69 ± 9	69 ± 10	64 ± 13	<0.001	69 ± 10	70 ± 10	69 ± 10	0.02	
CD19 (%)	10 ± 6	12 ± 6	12 ± 6	15 ± 14	<0.001	12 ± 6	12 ± 7	12 ± 9	0.99	

*Note:* Data are presented as the median ± interquartile range.

In CAD patients, CD19 was the only immune marker for which a significant progressive trend was observed with regard to serum vitamin D concentration ranges, with a greater proportion of C19-positive cells identified in patients with lower serum 25(OH)D_2_ levels (Table 4). In T2DM patients, the distribution of the percentages of CD8-positive cells, CD3-positive cells, CD56-positive cells, and the CD4/CD8 ratio differed significantly across the various serum 25(OH)D_3_ concentration ranges (Table 4), with the clear progressive trends in CD8 and CD4/CD8 suggesting the induction of a Th1 immune marker profile in 25(OH)D-deficient patients. Thus, T2DM patients might benefit from treatment with 25(OH)D_3_ dietary supplements.

In patients with hypertension, the percentage of cells with CD4, CD8, CD3 and CD19 as well as the CD4/CD8 ratio differed significantly across the various categories of serum 25(OH)D_3_ concentrations (Table 4). The clear trends in the CD4, CD8 and CD4/CD8 data suggested the induction of a Th1 immune marker profile in hypertension patients, with a greater proportion of CD8-positive cells observed in 25(OH)D_3_-deficient patients (<20 ng/mL), and a greater proportion of CD4-positive cells observed in patients with serum 25(OH)D_3_ >20 ng/mL. This finding is supported by the trends in CD3 and CD19, which suggest a transition from B-cell-mediated immunity to T-cell-mediated immunity, with progressively lower serum levels of 25(OH)D_3_. The percentage of CD3-positive and CD56-positive cells also differed significantly across the 25(OH)D_2_ concentration categories, but the lack of clear trends in CD3 and CD56 with regard to serum 25(OH)D_2_ concentration confounded our estimation of the effects of serum 25(OH)D_2_ levels on lymphocyte immune markers in patients with hypertension. Overall, these data suggested that patients with hypertension might benefit from dietary supplementation with 25(OH)D_3_.

## Discussion

Hypovitaminosis D is a global pandemic and is highly prevalent in older persons^49^, especially elderly women^51,52^. Previous studies have shown that a vitamin D deficiency is a risk factor for a number of immunity-related pathologies including autoimmune diseases^17,27,53^ and graft rejection^54,55^. Cell culture studies have shown that 1,25(OH)_2_D_3_ suppressed T cell proliferation, causing a shift in the T cell phenotype from Th1 to Th2^56^. Immunological studies in mice with experimentally-induced colitis showed that treatment with 1,25(OH)_2_D_3_ suppressed Th1 and Th17 cells and induced the differentiation of Th2 and T_reg_ cells^57,58^. Studies on experimental autoimmune encephalomyelitis in mice showed that 1,25(OH)_2_D_3_ induced T_reg_ cells^57,59–61^ and modulated the induction of Th17 T-cells by inhibiting the actions of IL-17^60,61^.

Hypovitaminosis D is also associated with a number of unrelated diseases including osteoporosis^53^, end-stage liver disease^62^, T2DM^63^, cancer^53^, obesity^9^ and CVD^64,65^, all of which are more prevalent in the elderly. However, whether vitamin D deficiency contributes to the pathology or progression of these diseases remains largely unclear^11^. Treatment with 1,25(OH)_2_D_3_ preferentially inhibits Th1 lymphocytes and induces Th2 cells^66–68^, as well as T_reg_ lymphocytes in the presence of IL-2^31,69^. Studies of pathogen-induced immunity suggest that the immune status of leukocytes *in vivo* is primarily driven by the local activation of vitamin D^70^. Therefore, the findings of *in vitro* studies using 1,25(OH)_2_D_3_ may not provide a completely accurate representation of the role of vitamin D in T-cell activation *in vivo*.

Among human leukocytes, CD8-positive lymphocytes have a relatively high density of vitamin D receptors^70,71^, and the primary effect of vitamin D treatment involves changes in gene expression^29,70^. Age has been shown to negatively impact T-cell clonal expansion and the repertoire diversity that can impair T-cell-mediated immune responses^72,73^. Studies of soluble proinflammatory factors and serum markers of oxidative stress have implicated low-level inflammation in the pathology of MetS, CVD^74^, T2DM^75^, obesity^76^, and hypertension^77^. Like hypovitaminosis D, these diseases are highly prevalent in the elderly^46^. Two recent studies of elderly persons in Europe with^37^ and without hypertension^36,78^ found that vitamin D deficiency was associated with an elevated serum level of the proinflammatory cytokine IL-6. However, the link between age-related changes in T-cell physiology and coinciding changes in the serum cytokine profiles of elderly people is unclear, and comprehensive studies of lymphocyte immune markers in patients with age-related diseases are scarce.

We investigated the relationship between serum vitamin D metabolites and lymphocyte immune markers in elderly patients in order to gain insights into the relationship between hypovitaminosis D and age-related diseases. Although the percentages of cells with CD4, CD8, CD3, CD19 and CD56 were within the normal reference range for Chinese patients^79,80^, serum levels of 25(OH)D_3_ and 25(OH)D_2_ were significantly correlated with three or more immune markers in CRVD, CAD, T2DM and hypertension patients (Table 2). Stratified analyses of the data showed that significant differences existed in the number of CRVD, CAD, T2DM and hypertension patients based on vitamin D metabolite levels, with the majority of such patients having serum levels of 25(OH)D_3_ and 25(OH)D_2_ that were ≤20 ng/mL and ≤2 ng/mL, respectively (Table 3). Clear, significant trends in immune marker percentages also existed with regard to serum vitamin D metabolite levels (Table 4). These trends suggested that vitamin D deficiency coincides with lymphocyte immune marker profiles representative of a shift from a Th2 to a Th1 immune response, with the strongest evidence observed in T2DM and hypertension patients.

Our findings regarding an increased percentage of CD8-positive cells in hypertension patients are consistent with the observation that the adoptive transfer of T-cells restored blood pressure regulation in a mouse model of hypertension, whereas the transfer of B-cells did not^81^. Studies of mouse models of obesity suggest that increased numbers of CD8-positive cells coincide with the recruitment and activation of macrophages in adipose tissue and the secretion of soluble proinflammatory factors associated with insulin resistance^82,83^, which is also consistent with our observation that the percentage of CD8-positive lymphocytes was increased in T2DM patients. The frequency of hypovitaminosis D observed in our elderly CRVD, CAD, T2DM and hypertension patients was similar to that reported by a study of the prevalence vitamin D deficiency among middle-aged and elderly people in Northwestern China^49^. Although the results of our stratified analysis based on serum vitamin D metabolite levels were not as convincing for CRVD and CAD as they were for T2DM and hypertension, our overall results suggest that CRVD, CAD, T2DM and hypertension patients might benefit from vitamin D dietary supplementation.

There are, however, certain limitations to our findings that should be considered in the interpretation of our results. Although the sample sizes of the CRVD, CAD, T2DM and hypertension groups were relatively large, our healthy control group was substantially smaller, and our observational analysis was limited to outpatient data recorded at a single hospital. In addition, we did not examine serum cytokine profiles in our patients, which could have provided further information regarding the Th2 to Th1 transition represented by the trends in CD8-positive cells and the CD4/CD8 ratio. Furthermore, we did not exclude patients receiving vitamin D dietary supplements or those whose lifestyle or dietary practices might have influenced their vitamin D status, all of which might have partially masked trends in the number of vitamin D-deficient patients in each group and the percentages of lymphocyte immune markers in the stratified analyses. In addition seasonal 25(OH)D_3_ changes have not been evaluated. Furthermore, we cannot exclude that different biologically active forms of Vitamin D, which have been discovered recently^84^, may have played a role since we only analyzed 25(OH)D_3_ and 25(OH)D_2_.

## Conclusion

**Figure 1:**
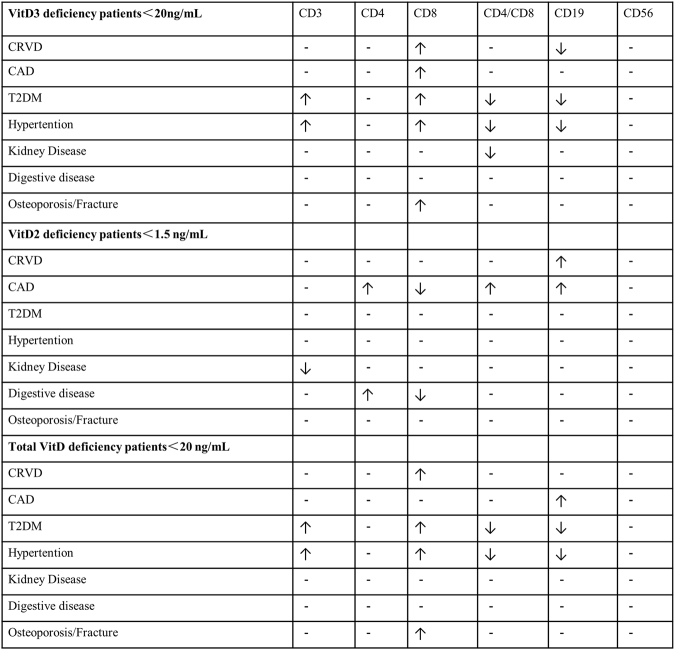
Summary graph of influence of Vitamin D deficiency on lymphocyte subsets in elderly patients with age-related diseases ↑, increase; ↓, decrease; -, no correlation.

In summary, we found a number of correlations between lymphocyte immune markers and vitamin D deficiency in middle-aged and elderly CRVD, CAD, T2DM and hypertension patients (Fig. 1), but future large-scale studies are warranted to confirm our findings.
